# Radachlorin-Containing Microparticles for Photodynamic Therapy

**DOI:** 10.34172/apb.2021.053

**Published:** 2020-08-17

**Authors:** Sergey Petrovich Krechetov, Anastasia Maksimovna Miroshkina, Maria Nikolaevna Yakovtseva, Elizaveta Nikitichna Mochalova, Andrey Vadimovich Babenyshev, Ivan Vladimirovich Maslov, Alexander Alexandrovich Loshkarev, Ivan Ivanovich Krasnyuk

**Affiliations:** ^1^Phystech School of Biological and Medical Physics, Moscow Institute of Physics and Technology, Dolgoprudny, Russia.; ^2^Department of Pharmaceutical Technology, First Moscow State Medical University, Moscow, Russia.; ^3^Center for Research on Molecular Mechanisms of Aging and Age-related Diseases, Moscow Institute of Physics and Technology, Dolgoprudny, Russia.; ^4^Phystech School of Electronics, Photonics and Molecular Physics, Moscow Institute of Physics and Technology, Dolgoprudny, Russia.

**Keywords:** Drug delivery systems, Microparticles, Radachlorin, Photodynamic therapy, Photosensitizing agents, Tumor cell line

## Abstract

***Purpose:*** Reducing the undesirable systemic effect of photodynamic therapy (PDT) can be achieved by incorporating a photosensitizer in microparticles (MPs). This study is devoted to the preparation of biocompatible biodegradable MPs with the inclusion of the natural photosensitizer Radachlorin (RС) and an assessment of the possibility of their use for PDT.

***Methods:*** RC-containing MPs (RС MPs) with poly(lactic-co-glycolic acid) copolymer (PLGA) matrix were prepared by a double emulsion solvent evaporation methods. The size and morphology of RC MPs were surveyed using scanning electron microscopy, confocal laser scanning microscopy, and dynamic light scattering. The content of RC, its release from RC MPs, and singlet oxygen generation were evaluated by the optical spectroscopy. Cellular uptake and cytotoxic photodynamic effect of RC MPs were investigated with *in vitro* assays.

***Results:*** The average diameter of the prepared RC MPs was about 2-3 μm. The RC MPs prepared by the water/oil/oil method had a significantly higher inclusion of RC (1.74 μg/mg) then RC MPs prepared by the water/oil/water method (0.089 μg/mg). Exposure of the prepared RC MPs to PDT light radiation was accompanied by the singlet oxygen generation and a cytotoxic effect for tumor cells. The release of the RC from the RC MPs was prolonged and lasted at least two weeks.

***Conclusion:*** PLGA RC MPs were found to cause a photoactivated cytotoxic effect for tumor cells and can be used for local application in PDT of tumors.

## Introduction

Chemotherapy is widely used in the treatment of cancer both as the main treatment method and as adjuvant therapy.^1,2^ Simultaneously, intensive work is carried out to create delivery systems based on microparticles (MPs) of micron and submicron (nanometer) sizes, which would enhance the efficiency of the impact of chemotherapeutic agents (CTA) in the treatment of cancer.^3,4^ When CTA-containing MPs are accumulated in the tumor, not only the local effect of CTA on molecular targets increases, but the undesirable adverse systemic effect of chemotherapy decreases.^4,5^ Furthermore, the drug resistance of tumors to CTA in a molecular form can be overcome when CTA is delivered to the tumor cell as part of MPs.^6^ Numerous studies demonstrate in the *in vitro* culture of tumor cells an enhanced damaging effect of MPs, which carry on the surface thereof molecular structures that recognize markers of target cells.^7^ However, the absence of appropriate markers in the capillary endothelium makes it inefficient to accumulate such tumor-specific MPs in the tumor when they are injected into the bloodstream *in vivo*.^8-10^ Insufficient selectivity of MPs accumulation in the tumor, when administered to the bloodstream in actual practice, is overcome by their local administration to the tumor or by arterial embolization of the problem area.^11,12^

The use of photosensitizers (PS) as CTA allows to reduce the systemic side effects of chemotherapy by localization of cytotoxic effect in the tumor when it irradiated by the light of a certain wavelength.^13,14^ Local use of the photodynamic therapy (PDT) procedure reduces systemic side effects when PS is administered to the systemic bloodstream but does not completely exclude them.^15^ The inclusion of PS in the composition of MPs allows improving the efficiency of PTD.^16^ Besides, the inherent fluorescence of PS allows visualizing the MPs with these agents in the body’s tissues. This makes MPs with PS suitable for theranostics: diagnostics, treatment, and monitoring of changes in the tumor during the treatment.^17^

Radachlorin^Ò^ (RC) is a natural second-generation PS used for fluorescent diagnostics and PDT of malignant tumors.^18^ It comprises sodium salts of chlorin e6 (up to 90%), chlorin p6, and purpurin 5 as active agents. The presence of a pronounced maximum in the red region of the absorption spectrum (662 nm) and a high quantum yield of the singlet oxygen generation when light is absorbed in this region enables high phototoxicity of the RC in vivo and in vitro.^19,20^ Selectivity of RC accumulation in the tumor and its rapid clearance determines the low sensitivity of patients’ skin to daylight and a small number of systemic side effects after PTD.^18,20^ The efficiency of PDT using RC is shown for malignant neoplasms of the skin, lungs, gastrointestinal tract, and genitourinary tract.^21-23^ The presence of inherent fluorescence in RC, when exposed to light, used in PDT,^19^ makes it possible to use this PS for both PDT and fluorescent diagnostics.^22,23^

The inclusion of RC into MPs is considered as a promising trend for improving the efficiency of its use for PDT and fluorescence diagnostics.^24^ The preparation of such MPs should potentiate intracellular accumulation and deposition of RC, as well as prolongation of its effect. This will allow further localize the effect of this PS and to extend the duration of the therapeutic window for PDT. Therefore, the objective of this work was to prepare biocompatible biodegradable polymer MPs with the inclusion of RC and evaluation of the possibility of their use for PDT.

## Materials and Methods

### Materials

For preparing RC-containing MPs (RC MPs) as a source of PS was used a concentrate for the infusion solution “Radachlorin^Ò^” manufactured by RADA-PHARMA (Moscow, Russia), comprising 3.5 mg/mL of a mixture of sodium salts of chlorin e6, chlorin p6 and purpurin 5. Poly(lactic-co-glycolic acid) 65:35 copolymer (PLGA) RESOMER RG 653 H was purchased from Evonik Industries AG (Essen, Germany); span 80, Polysorbate 20, light mineral (paraffin) oil, 1,3-diphenylisobenzofuran (DPBF) were from Sigma (Saint Louis, MO, USA); polyvinyl alcohol 18-88 was from Merck (Darmstadt, Germany); methylcellulose A4M was from Ashland (Covington, KY, USA); DMEM12 medium, penicillin, streptomycin, L-glutamine, 3-(4,5-Dimethyl-2-thiazolyl)-2,5-diphenyl-2H-tetrazolium bromide (MTT) were from PanEco (Moscow, Russia); fetal bovine serum was from Hyclone (Chicago, IL, USA). Methylene chloride, acetonitrile, isopropyl alcohol, n-hexane, n-heptane, as well as the reagents used for preparing buffer solutions, were qualified as chemically pure or pure for analysis and were obtained from Chimmed (Moscow, Russia). Experiments were conducted using purified water obtained by a reverse osmosis UVOI 1812C6 system (MEDIANA FILTER, Moscow, Russia).

### Preparation of RC MPs

For the preparation of RC MPs by multiple water-in-oil-in-water (W/O/W) emulsion method, RC was precipitated from 200 ml of the concentrate for the infusion solution by adding 400 ml of 0.1 M HCl followed by centrifugation for 10 minutes at 1500 g. The wet precipitate of RC was dispersed in an ice bath in 2 ml of a 2.5% PLGA solution and 0.5% span 80 using SONOPULS HD 2070 ultrasonic homogenizer (Bandelin, Germany) with an MS 73 micro emitter at 15% power for 1 minute with a 10% pulsation. The resulting primary water-in-oil emulsion was dispersed by adding dropwise to 20 ml of an aqueous solution of 1% polyvinyl alcohol and 0.5% methylcellulose at a temperature of 4°C using Ultra Turrax 25 (IKA, Germany) with an S 25 N – 8 G dispersant at 20 000 rpm. The resulting secondary W/O/W emulsion was mixed with 500 mL of water, cooled down to 4°C, and kept for 6 hours at room temperature, stirring it at 200 rpm with a Eurostar 20 digital overhead *stirrer*(IKA, Germany) with a propeller stirring element to remove dichloromethane and solidify MPs. The maturated MPs were separated by centrifugation for 20 minutes at 150 g. The precipitate was washed by resuspension in 15 mL of water, followed by centrifugation thereof under the above-said conditions. The described washing of MPs from the components of the secondary dispersion medium was performed three times. The washed MPs were frozen in liquid nitrogen and freeze-dried in a VaCo 2 lyophilizer (ZIRBUS, Germany).

For the preparation of RC MPs by multiple water-in-oil-in-oil (W/O/O) emulsion method, RC was precipitated as described above for the W/O/W emulsion method. The precipitate was dispersed in 0.5 mL of acetonitrile by intensive shaking until it became homogeneous, and then mixed with 1 mL of 5% (weight/volume) PLGA solution in acetonitrile and obtained a weakly opalescent water-in-oil dispersion of RC. The resulting primary dispersion was added dropwise to 20 ml of a 5% (weight/volume) span 80 solution in mineral oil and emulsified using Ultra Turrax 25 with an S 25 N – 8 G dispersant at 22000 rpm. 10 ml of n-heptane was added to the resulting secondary W/O/O emulsion and further homogenized for 10 minutes, and then another 10 mL of heptane was added and homogenized for another 30 minutes. The resulting W/O/O emulsion was kept for 6 hours at room temperature, constantly stirring it with an overhead stirrer with a propeller stirring element at 500 rpm. After that, the emulsion was left overnight in a fume cupboard without stirring. The maturated MPs were separated by centrifugation for 20 minutes at 150 g. To wash the separated MPs free from the mineral oil and emulsifier 20 mL of a mixture of isopropanol – heptane (1:3, by volume) was poured to it, stirred for 5 minutes at 700 rpm, and centrifuged under the above-said conditions. The described washing was performed three more times. The washed RC MPs were dried for 1 hour at a vacuum of 2 mbar (PC 3001 VARIO pump, Vacuubrand, Germany), and then kept for 1 hour at a vacuum of 0.5 mbar (EVD6 pump, Eurovacuum, Germany) for removal of solvents.

### Characterization of RC MPs

The morphology and sizes of the RC MPs were evaluated using scanning electron microscopy (SEM) images obtained using a MAIA3 microscope (TESCAN, Czech Republic). The samples of MPs for electron microscopy were applied onto silicon substrates as a suspension in water and dried in air at room temperature. The measurements were made at an accelerating voltage of 1-2 kV. To determine the sizes of MPs, digital microphotographs were processed using ImageJ 1.52a program. The distribution of RC in MPs was evaluated by the images of MPs in an aqueous suspension prepared using a confocal laser scanning microscope LSM 780 (Carl Zeiss, Germany).

The charge of the RC MPs surface was characterized by their zeta-potential in the aqueous suspension, which was measured using a dynamic light scattering (DLS) apparatus Zetasizer Nano ZS (Malvern Instruments, USA) at a temperature of 25^o^C.

To determine the content of RC, a weighed amount of RC MPs was dissolved in acetonitrile, and the optical density was measured at 664 nm against the solution in acetonitrile of the same weighed amount of PLGA. As a calibration, we used solutions of RC in acetonitrile prepared by diluting the concentrate for the infusion solution. The water content in the calibration solutions was less than 0.05% by volume. The measurements of the optical density and optical absorption spectra of the samples were measured using a UV 3600 spectrophotometer (Shimadzu, Japan).

### Release of RC from RC MPs

The release of RC from MPs was studied in a 50 mM potassium phosphate buffer having a pH of 7.4. 10 mg MPs was suspended in 5 mL of the buffer in a centrifugal test-tube, and incubated in a thermostat at 37^o^C. Before the collection of samples for determining the released RC, the suspension in the test-tube was mixed by shaking until it became homogeneous, and then MPs were separated by centrifugation at 500 g for 5 minutes. For each time, 0.3 ml of the supernatant was selected, and the precipitate was resuspended in the remaining volume of the supernatant. The collected samples were stored frozen at a temperature -20^o^C. At the end of the experiment, all the collected samples were defrosted, and the concentration of RC was determined by its fluorescence using a CLARIOstar Plate Reader (BMG LABTECH, Germany) in 96-well plates. Fluorescence was excited at a wavelength of 401 nm and registered at a wavelength of 660 nm. For calibration, we used solutions of RC in the described potassium phosphate buffer prepared by diluting the concentrate for the infusion solution. The release of RCfrom MPs was compared with its destruction during the incubation under the same conditions.

### Generation of singlet oxygen by RC MPs

The ability of RC in the composition of MPs to intensify the generation of singlet oxygen under conditions of PDT was evaluated in the course of irradiation with light from a light-emitting diode (LED) LZ100R202 (OSRAM SYLVANIA LED Engin, USA) with maximum radiation at 660 nm. 2 mL of a suspension comprising 5 mg MPs in a 50 mm potassium phosphate buffer with a pH of 7.4 with 0.5% Polysorbate 20 was placed in a standard 1 cm x 1 cm × 4 cm spectrophotometer cuvette at a temperature of 25^o^C. 20 µL of 1 mM of DPBF solution in ethanol was added to the suspension, and then the cuvette was irradiated for a preset time from the top by LED light with the 5 mW/cm^2^ irradiance on the surface of the suspension. Before and after irradiation, the optical density was measured at 415 nm in the absorption band of DPBF, converting during the interaction with singlet oxygen into endoperoxide, not absorbing at this wavelength.^25^ Loss in the optical density was used as a characteristic of the intensity of the singlet oxygen generation. The generation of singlet oxygen by RC MPs was compared with the generation of singlet oxygen in the solution of RC. In this instance, instead of the MPs suspension, 2 mLof 8.7 µg/mL of the solution of RC in the said potassium phosphate buffer was placed in the cuvette of the spectrophotometer. The generation of singlet oxygen after the dissolution of MPs and RC in acetonitrile was measured in the described way, with the difference that the same volume of this organic solvent was used instead of potassium phosphate buffer.

### Study of the photodynamic effect of RC MPs in cell culture

The cell cultures of CHO (gold hamster ovarian cells), MCF-7 (human mammary adenocarcinoma cells), and B16 (mice melanoma cells) were obtained from the Russian collection of vertebrate cell cultures (Institute of Cytology of the Russian Academy of Sciences, Saint Petersburg). 6-well plates were used to study the effect of MPs on cells. 10^5^ cells were seeded into a well in 2 mL of DMEM12 medium comprising 10% fetal bovine serum, 100 IU/mL of penicillin, 0.1 µg/mL of streptomycin, and 2 mM of L-glutamine. The cells were cultured in the dark at a temperature of 37°C in humidified air with 5% CO_2_ content. After 24 hours of cultivation and formation of a monolayer for each cell line, in one-third of the wells the culture medium was replaced by the fresh one, in another third of the wells - with 0.5 mg/mL MPs suspension in the culture medium, and in the last third of the wells - with 1.75 µg/mL of the RC solution in the culture medium. 24 hours later, the culture medium was replaced with the fresh one in all the wells, and half of the wells of each type were irradiated for 1 minute with LZ1-00R202 LED light, with 25 mW/cm^2^ irradiance on the surface of the culture medium. The effect of MPs and photodynamic irradiation on cells was evaluated after 24 hours.

### Microscopy of cells and cytometric analysis

Microphotography of cells was performed using an Axio Observer A1 light microscope (Carl Zeiss, Germany) equipped with Hal 100, HBO 100 illuminators, and Axiocam 503 mono digital monochrome camera controlled by ZEN 2 software. A light filter unit A 45 HQ TexasRed (EX BP 560/40, BS FT 585, EM BP 630/75) was installed during microscopy.

The cytometric analysis was performed on an Amnis ImageStream X Mark II imaging flow cytometer (Luminex Corporation, USA) using a 40× objective lens. A 785 nm (0.05 mW) laser was used to measure side scattering, and 488 nm and 642 nm (200 and 150 mW, respectively) were used to excite fluorescence. 6000 events were collected for each sample. The received data were processed using the IDEAS software.

### Cell viability assay

To assess cell viability in the MTT test,^26^ the culture medium in all the wells was replaced with the fresh one, comprising 0.5 mg/mL of MTT, and it was incubated for 0.5 hours in the dark at a temperature of 37°C under an atmosphere with 5% CO_2_. After that, the medium was removed and the content of the wells was dissolved in 1 mL of dimethyl sulfoxide. The amount of the resulting formazan was characterized by the optical density of the prepared solutions in the wells at 550 nm, measured using a CLARIOstar plate reader. Cell viability after exposure to MPs and photodynamic irradiation was characterized by the ratio of the optical densities in the wells with the exposed cells, and the wells with the control cells of the same cell line without any exposure (100% viability).

### Statistical analysis

Statistical analysis of experimental data was performed using the Microsoft Office Excel program. The data are presented as mean and standard deviation according to the results of at least three independent measurements. The significance of differences was evaluated by the Student *t* test.

## Results and Discussion

### Characteristics of RC MPs

**Figure 1:**
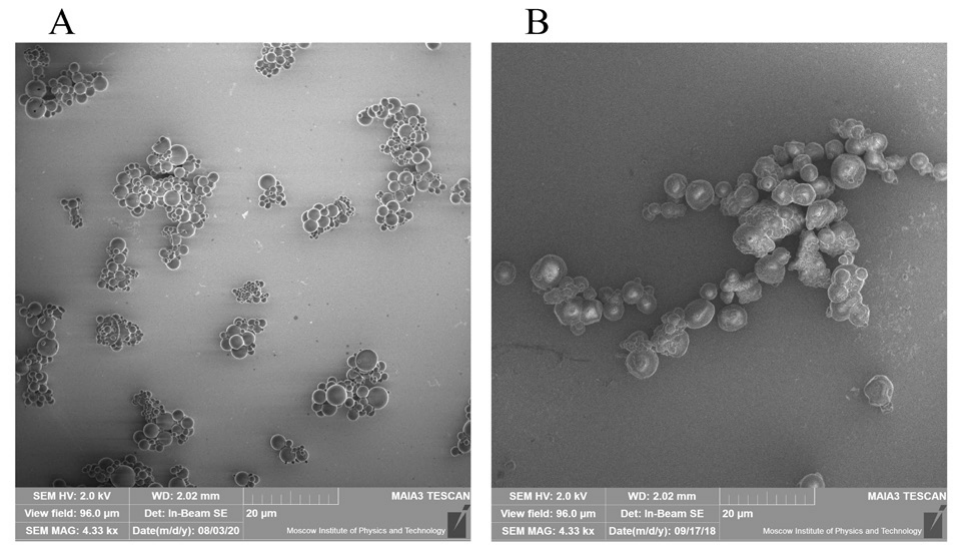
SEM of RC MPs

**Figure 2:**
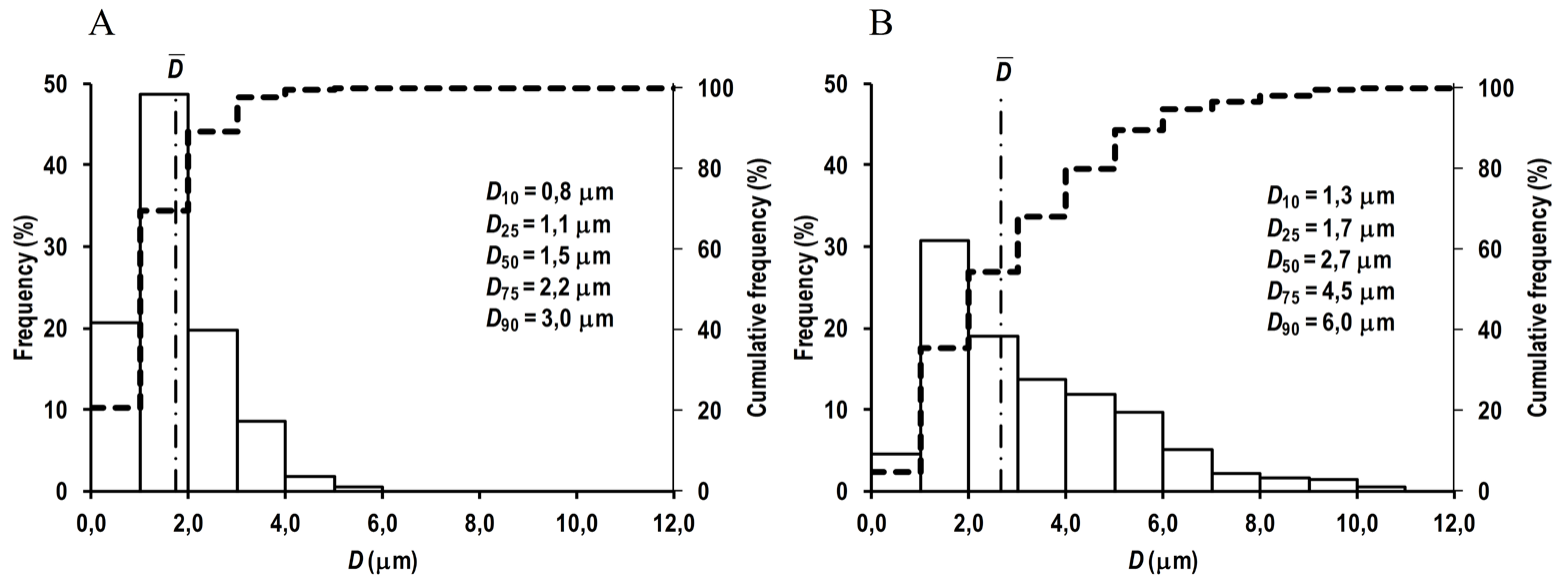
Density histogram (solid line) and cumulative distribution histogram (dashed line) of RC MPs diameters.

The prepared RC MPs, according to the SEM images (Figure 1), have a spherical shape and are prone to form agglomerates both when prepared by method W/O/W and by method W/O/O. Taking into account the formation of agglomerates, the RC MPs size was measured by the obtained SEM images, since when measured using DLS, the agglomerates act as integral particles and their presence substantially distorts assessment of the sizes. According to the obtained data, the most amount of RC MPs prepared by both methods have the size (Figure 2) up to 10 µm (*D*_90_ < 10 µm). The RC MPs prepared by method W/O/O are larger and have a greater polydispersity than the RC MPs prepared by method W/O/W (Table 1). The presence of terminal carboxyl groups in the PLGA used for preparing RC MPs determined a negative charge of the prepared MPs in water (Table 1). The obtained value of zeta-potential ranging from -30 mV to -40 mV is at the low level of the values known from the literature for unmodified MPs,^27^ which indicates the absence of a noticeable effect of RC in the composition of MPs on their charge.

(A) RC MPs prepared by method W/O/W, (B) RC MPs prepared by method W/O/O.

(A) RC MPs (n = 1274) prepared by method W/O/W, (B) RC MPs (n = 661) prepared by method W/O/O.

**Table 1:** Characteristics of RC MPs

**Method** **of MPspreparation**	Diameter of MPs, µm ()*	**Polydispersity index** ()*	**Zeta-potential of MPs, mV**	**Content of** **RC in MPs, µg/mg**	**Loading efficiency of** RC in MPs**, %
W/O/W	1.74 ± 0.91	0.27	-38.6 ± 6.3 *n* = 9	0.089 ± 0.007	0.8 ± 0.1
W/O/O	3.28 ± 2.00	0.37	-33.8 ± 3.7 *n* = 15	1.74 ± 0.21	12.6 ±1.5

Note. * - values are calculated from the results of particle size measurement on SEM images, ** - the percentage of loading efficiency was calculated as the percentage of content of RC in RC MPs relative to its theoretical content under the condition of its full inclusion.

**Figure 3:**
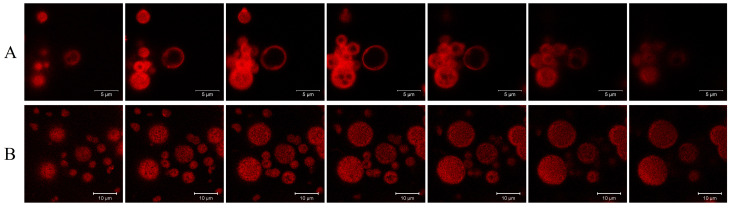
Images of confocal microscopy optical z-sections of RC MPs.

Confocal microscopy showed the presence of non-fluorescent zones occupying a noticeable part inside the RC MPs prepared by method W/O/W (Figure 3A), while the internal structure of the RC MPs prepared by method W/O/O was characterized by a uniform distribution of fluorescent and non-fluorescent small regions (Figure 3B). The described picture, seemingly, reflects the particularities of the primary dispersion W/O, which has a different dispersion medium in the methods used for preparing MPs. In method W/O/W, the primary dispersion medium is the PLGA solution in methylene chloride, mutual solubility of which with water is insignificant. Therefore, the resulting dispersed system is an emulsion with droplets of an aqueous concentrate of RC in methylene chloride. The amphiphilicity of chlorins determines the partial dissolution of RCin methylene chloride.^28^ During the maturation of RC MPs after the preparation of a secondary emulsion W/O/W, RC dissolved in methylene chloride is fixed in the polymer matrix, but RC remaining in the water droplets can be washed out through the openings that emerge on the surface, which explains the presence of non-fluorescent zones, which do not contain RC, in RC MPs prepared by method W/O/W. In method W/O/O, the primary dispersion medium is the PLGA solution in acetonitrile, freely mixing with water. The primary W/O dispersion prepared in this instance is characterized by high dispersion of precipitate RC (without opalescence and formation of a precipitate after centrifugation for 10 minutes at 1500 g) and, possibly, by the dissolution of chlorins in the resulting primary dispersion medium. Diffusion of acetonitrile into a secondary dispersion medium during the maturation of RC MPs after preparing a secondary W/O/O emulsion results in further aggregation of particles of precipitate RCand fixation thereof in a weakly fluorescent polymer matrix in the form of fluorescent regions.

(A) RC MPs prepared by method W/O/W, (B) RC MPs prepared by method W/O/O. Fluorescence was excited by a 405 nm laser and total fluorescence emission was registered in the wavelength range from 597 nm to 695 nm. The step of the optical z-sections is 1 μm.

The proposed description of the structure formation during the maturation of RC MPs is consistent with the evaluation results of the RC inclusion therein (Table 1). Thus, the inclusion of RC in the MPs prepared by method W/O/W is very low and is less than one percent of the maximum possible. This is substantially lower than the inclusion of RC in the MPs prepared by method W/O/O when the inclusion is by order of magnitude greater. The obtained result is well explained by the washing of RC out from the cavities found in the W/O/W RC MPs and absent in the W/O/O RC MPs. At the same time, it is noteworthy that the loading efficiency of RC in the W/O/O RC MPs is not high and has a value slightly exceeds 10%. The reason for this can be the observed amphiphilicity of chlorins,^28^ which may result in the release of part of RC from the maturing RC MPs with dissolution in a secondary oil dispersion medium and embedding in the reverse micelles formed by Span 80.^29^

### Photodynamic properties of RC MPs

**Figure 4:**
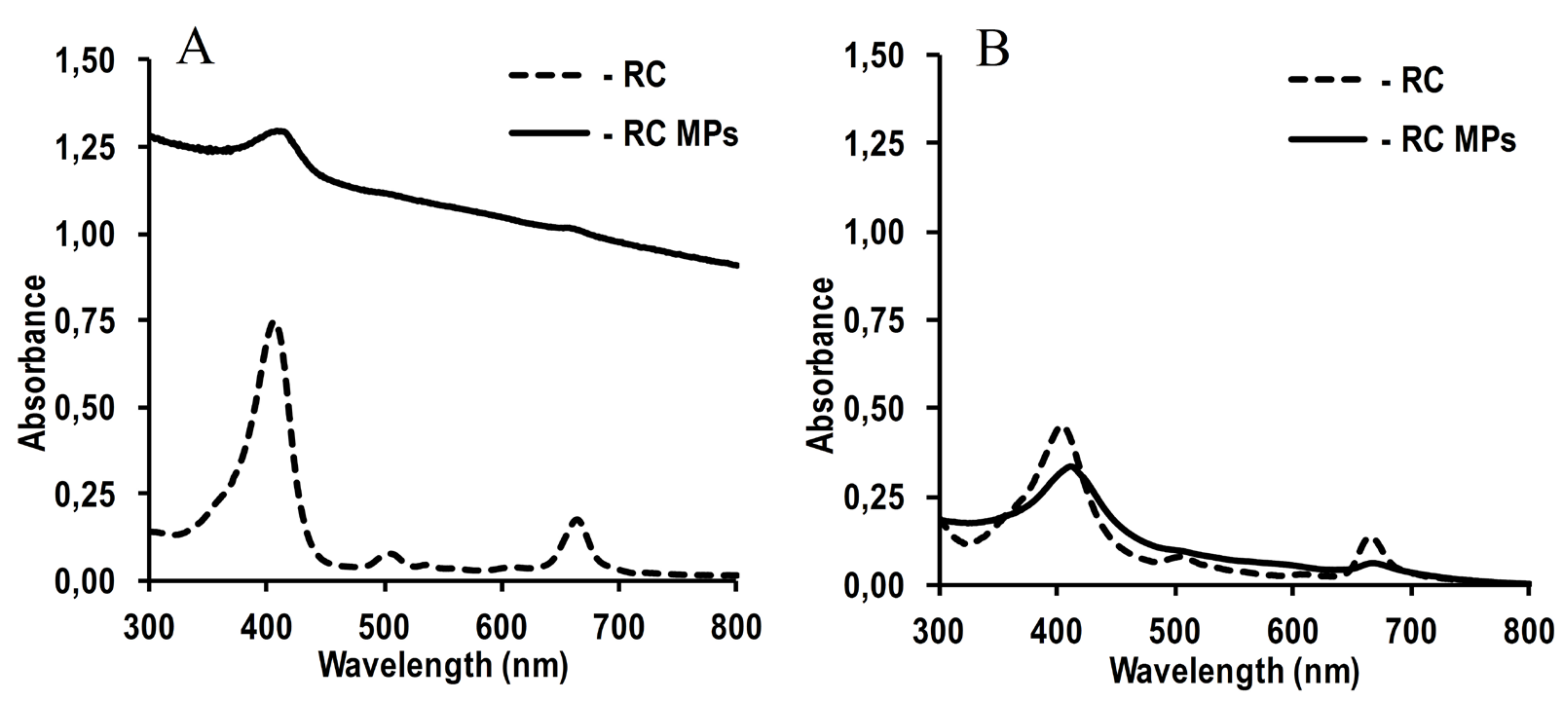
Absorption spectra of RC MPs (2.5 mg/ml) prepared by method W/O/O and RC (8.75 μg/ml).

**Figure 5:**
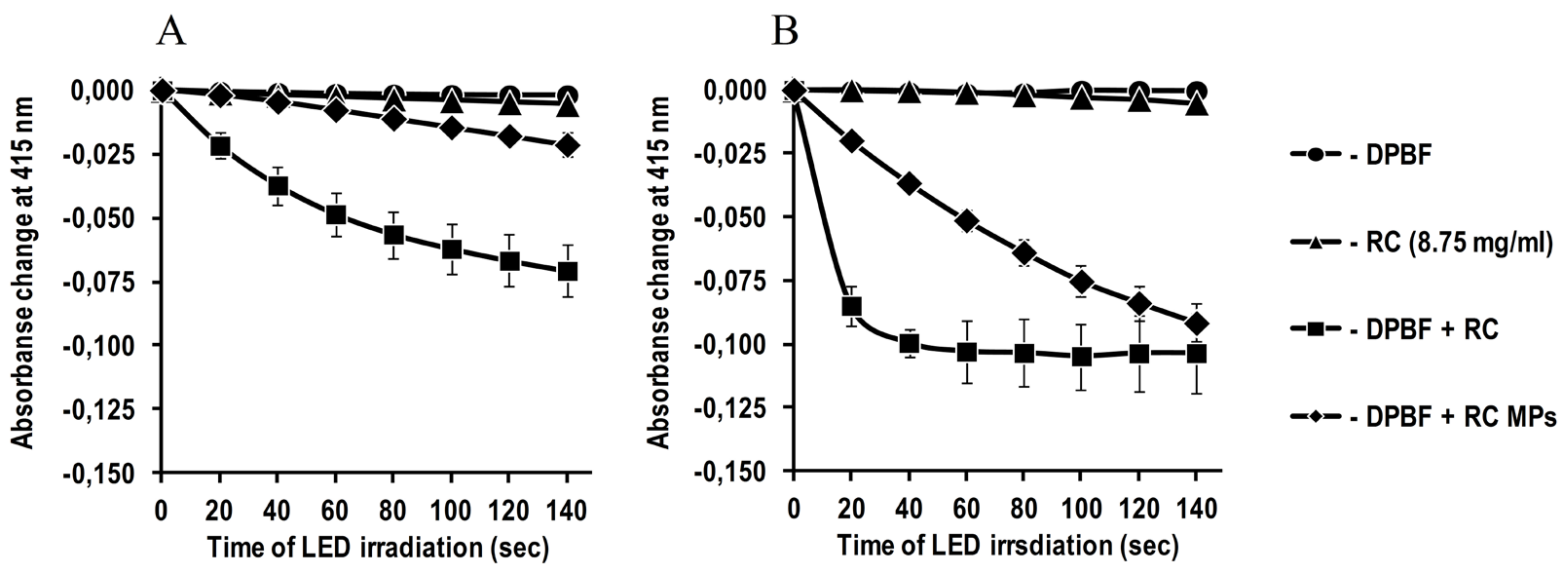
Generation of singlet oxygen by RC MPs (2.5 mg/ml) prepared by method W/O/O and RC

The absorption spectra of an aqueous suspension of RC MPs prepared by method W/O/O and a solution of these MPs in acetonitrile show characteristic peaks of RC (Figure 4): long-wavelength at about 660 nm and short-wavelength at about 400 nm. This indicates that RC, encapsulated in MPs, retains the original optical properties that are important for PTD.^30^ Although the featureless of pronounced peaks reflects a low content of RC in MPs, however, it is sufficient to generate singlet oxygen (Figure 5). The detected significant increase in the singlet oxygen generation during irradiation of an aqueous suspension of RC MPs by the red LED with emission in the region of an absorption long-wavelength band (about 660 nm) of RC (Figure 5A) indicates that they can be used for PDT.

(A) 50 mM potassium phosphate buffer with pH 7.4, (B) acetonitrile.

(8.75 μg/ml) during LED irradiation with λmax = 660 nm.

(A) 50 mM potassium phosphate buffer with pH 7.4, (B) acetonitrile. The decrease of absorption at 415 nm is shown as dependence on irradiation duration.

**Figure 6:**
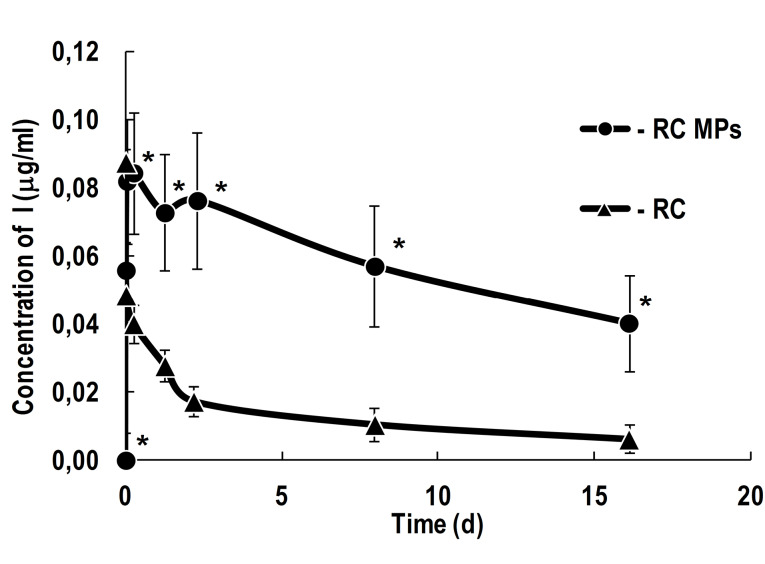
Release of RC from RC MPs prepared by method W/O/O in comparison with the change of concentration of free RC in 50 mM potassium phosphate buffer with a pH 7.4.

The observed generation of singlet oxygen in the presence of RC MPs in the aqueous medium seems to be mainly associated with the release of RC into the ambient medium. According to the obtained data (Figure 6), during the first hour in the aqueous medium maximum concentration ofRC is achieved, which then slowly decreases for more than two weeks. Under the same conditions, the concentration of RC in the solution without MPs decreases much faster and, after a day, it is significantly lower than in the RC MPs suspension, and after a few days it becomes lower than 10% compared to the initial concentration. The concentration of RC in the RC MPs suspension decreases only to half of the maximum concentration after two weeks only. The described release kinetics characterizes RC MPs as a depot with prolonged release of RC, suitable for performing several consecutive PDT sessions without repeated injections of a PS.

The initial concentration of RC in the solution without particles is 0.0875 μg/ml. * - *P* < 0.05 for the differences from samples with RC alone.

The obtained kinetics of RC release reflects the strong effect of the drug destruction on its total concentration in the aqueous medium surrounding RC MPs. This results in a descending release profile that is not described by conventional models.^31-33^ Such kinetics requires the development of a special model that takes into account the destruction of the released drug. However, the use of a model that takes into account the destruction of only the released drug in the case of RC may not be enough.^34^ The penetration of water into gradually swelling and disintegrating MPs, before the release of the drug, can lead to the destruction of RC before its release into the environment and exert an influence on the result of determining the cumulative release.^35^

### Impact of RC MPs on tumor cells in culture

**Figure 7:**
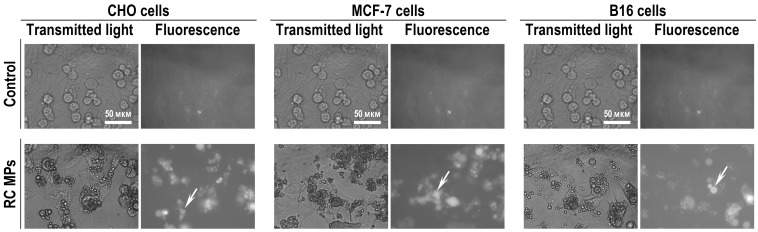
Transmitted light and fluorescence microscopy of the cell’s monolayers after a 1-day incubation with RC MPs prepared by method W/O/O.

**Figure 8:**
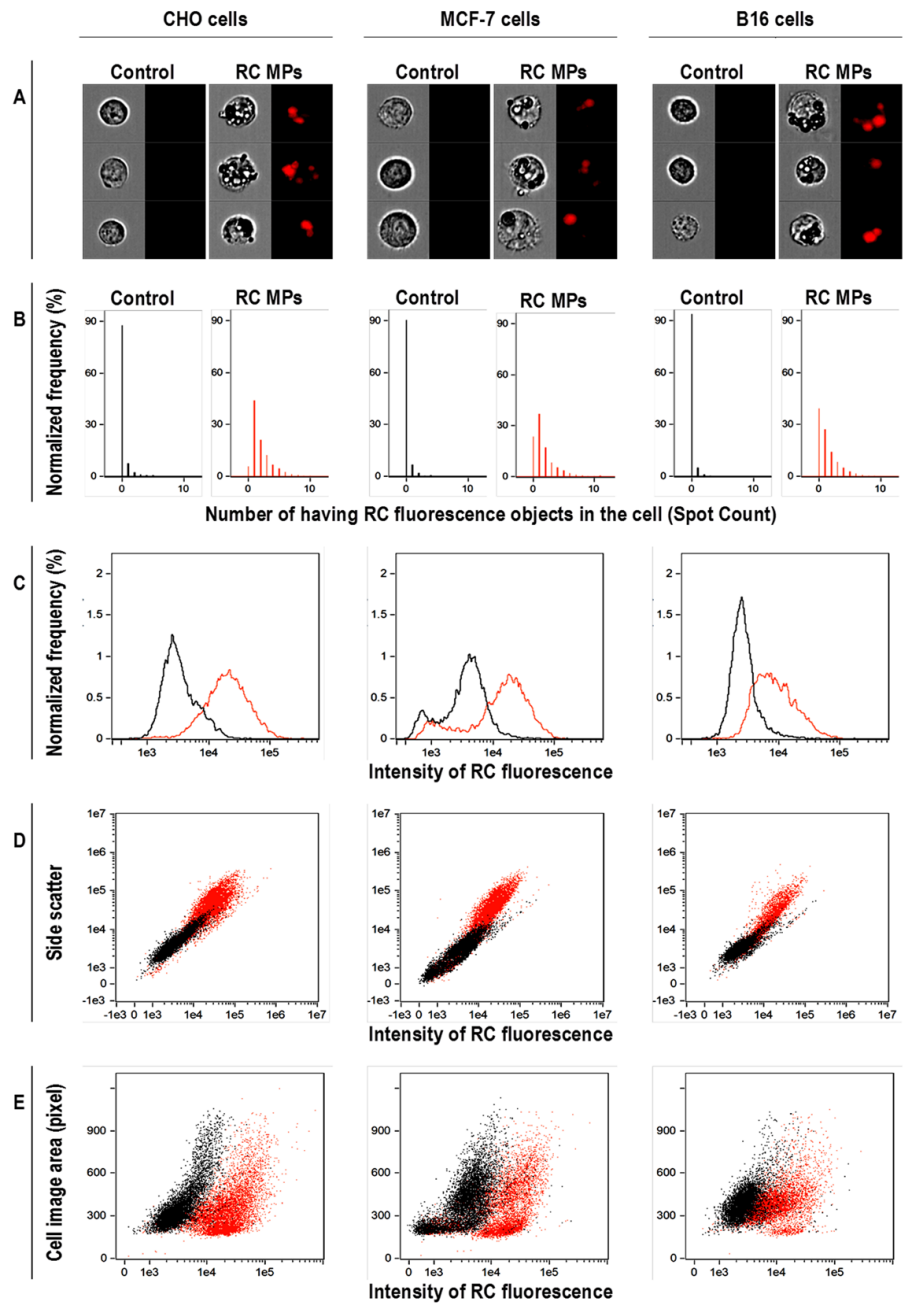
Imaging flow cytometry data of cells after a 1-day incubation with RC MPs prepared by method

According to the results of the studies using light microscopy and imaging flow cytometry, RC MPs are internalized by the transformed cells of the CHO, MCF-7, and B16 lines in culture. The images of light microscopy (Figure 7), obtained a day after adding RC MPs, show that RC MPs are located inside the cells, and no significant changes in the density of the monolayer occur. After the cells are transferred to the suspension and washed, imaging flow cytometry also characterizes the RC MPs position as intracellular (Figure 8). Imaging flow cytometry is widely used to study the interactions of liposomes, magnetic nanoparticles, gold nanoparticles, etc. with cells.^36-39^ Data presented in Figure 8 were obtained using Amnis ImageStream X Mark II imaging flow cytometer. ImageStream, unlike conventional cytometers, employs in its optical systems a time-delay integration-CCD camera,^40^ which takes photos of objects in the flow instead of detection of scattered light. Thus, the cytometer allows the evaluation of morphology and direct localization of the fluorescent and side scatter signals in individual images.^41^ In the (Figure 8A) the location of RC MPs inside the cell contour is visible. In our study using IDEAS analysis software provided with the cytometer, we applied the Spot Count feature to images of cells preincubated with RC MPs (Figure 8B). The feature provides the number of objects with RC fluorescence on cells using the connected component algorithm which examines the connectivity of each pixel to a particular spot or to the background.^42^ As a result, a histogram was plotted indicating the fraction of the total number of images (in percent) with the corresponding number of spots.Determination of the number of objects with fluorescence RC in the cell images as an indicator of the RC MPs internalization by the cells shows that after the incubation with RC MPs,^43^ the images of at least 90% of CHO cells, 80% of MCF-7 cells, and 60% of B16 cells feature from one up to several spots that fluoresce as RC MPs. The significant shift to the right in the distribution of the fluorescence intensity of RC in the cells incubated with RC MPs compared to the intact cells (Figure 8C) confirms the association of RC MPs with the cells. The intracellular presence of RC MPs is indicated by the increase of side light scattering of the cells proportional to the increase in fluorescence RC after the incubation with RC MPs (Figure 8D) in parallel with the minor changes in the cells size (Figure 8E).

Examples of RC MPs inside the cells are indicated by arrows.

W/O/O.

(A) Microphotographs of imaging flow cytometry with transmitted light (left) and fluorescence (right), (B - E) results of flow cytofluorometry. On the diagrams the data of cells after the incubation with RC MPs are red and the data of control cells are black.

**Figure 9:**
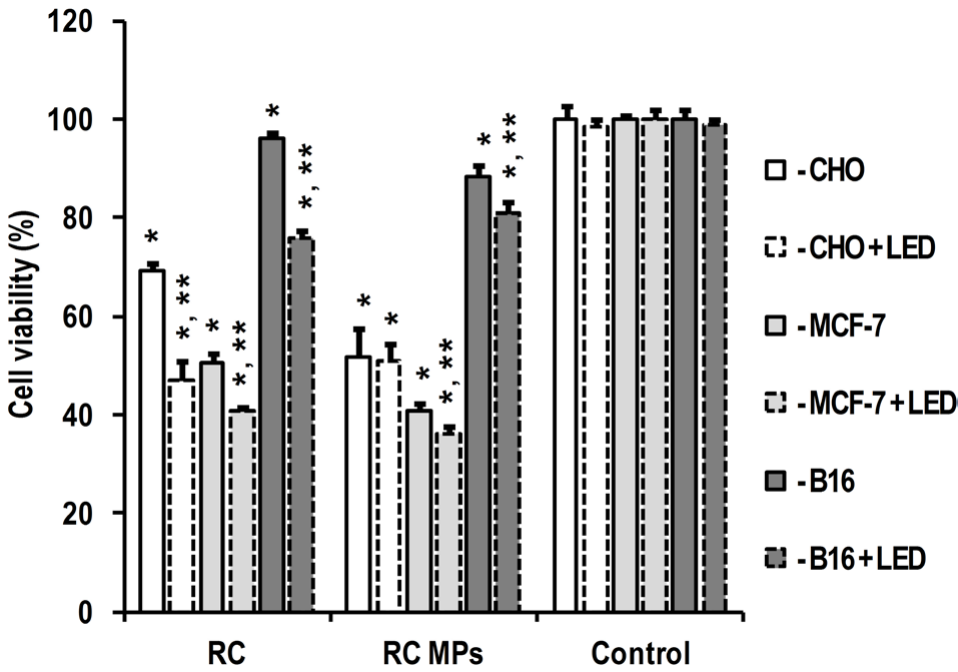
Effect of RC and RC MPs prepared by method W/O/O on cells viability and photodynamic effect of light irradiation according to MTT-test results.

According to the results of the MTT test, the internalization of RC MPs by cells in culture is accompanied by a decrease in their viability (Figure 9). The observed inhibition of metabolism can be associated with both the cytotoxic effect of RC released inside the cells and with the disturbance of their structure by the internalized RC MPs.^44^ Similarity of interline ratios of the intensity of changes in cell viability caused by RC MPs to the changes caused by RC indicate the similar nature of the disturbing factor in both instances. This means that the leading role in the occurring inhibition of cell viability is played by the release of RC from RC MPs. Exposure to red light in the absorption band of RC is accompanied by a further decrease in the viability of the cells incubated with RC MPs (Figure 9). The observed photodynamic effect in the presence of RC MPs is significant in MCF-7 and B16 cells, but not significant in CHO cells. Although the photodynamic effect of RC without MPs is more pronounced, its presence in the samples with RC MPs indicates the applicability of such MPs in PDT.

* - *P* < 0.05 for differences from same line cells without any additives and irradiation, ** - *P* < 0.05 for differences from same line cells under the same conditions without LED irradiation.

Thus, according to the obtained experimental data, the use of the W/O/O technology makes it possible to prepare RC MPs suitable for use as a depot, which for at least two weeks can maintain in its microenvironment the PS concentrations capable of causing a cytotoxic photodynamic effect. When the prepared RC MPs are delivered to the tumor, their absorption by cells will increase the PDT efficiency due to the intracellular release of the active agent. Since RC MPs have a size ranging from 1 to 10 µm and a negative charge, their absorption by cells should be similar to the phagocytosis type. The maturation of endosomes in this case involves their fusion with lysosomes and is accompanied by acidification of the internal environment up to a pH 4.5. With this pH, the destruction of MPs from PLGA is insignificantly accelerated,^45^ but the solubility of RC in water considerably decreases, and its lipophilicity increases.^46^ This, in combination with limited exocytosis of the endosome content after fusion with lysosomes, is a prerequisite for reliable retention of released RC in tumor cells.

In the case of intravascular administration, the accumulation of the prepared RC MPs is most likely to occur mainly in the liver, spleen, lungs, and other organs with a high content of cells of the mononuclear phagocyte system. The yield of the prepared RC MPs from the bloodstream in other locations will be low due to a small portion of submicron-sized MPs that are actively captured by endothelial cells and undergo transcytosis.^47^ Due to the relatively large size of the prepared RC MPs, their retention in the altered tumor vessels should be insignificant.^10^ At the same time, the diameter of the prepared RC MPs is smaller than the diameter of the MPs used for the embolization of the problem area. Given the diameter of the terminal arterioles of 10-50 µm and capillaries of 8-10 µm, it is expedient to use MPs with a diameter of at least 40 µm.^48^ Therefore, the prepared RC MPs, the basic size of which is less than 10 µm, are not applicable for transarterial embolization of tumors. Thus, the accumulation of such RC MPs in the desired part of the circulatory system requires giving RC MPs properties leading to their retention on the endothelial surface of the problem area. This aim can be achieved by binding specific endothelial markers of the problem area with fixed on the surface of RC MPs antibodies.^49^ Another way of accumulating RC MPs in the desired region of the body may be the effect of an external physical field that interacts with sensitive to this field nanoparticles, which are included in the MPs matrix.^50^

The successful use of PTD with RC for antibacterial therapy and the experimental data regarding the efficiency of antibacterial PDT using MPs allows considering the possibility of using theRC MPs prepared in this work in the composition of local antibacterial photodynamic prolonged-release drugs.^51,52^ In the long-term treatment of chronic infectious processes, the use of such drugs will help to reduce the frequency of medication of the infected sites and will be accompanied by an absence of systemic side effects, and, as distinct from antibiotics, will not be accompanied by the suppression of the patient’s native microflora beyond the bounds of the treated area. The mechanism of antibacterial action of RC, as well as of other PS, in PDT is not specific to the type of pathogenic microorganisms and does not imply the development of resistance thereof to this type of antibacterial therapy.

## Conclusion

Generally, the obtained results demonstrate that based on the biodegradable PLGA it is possible to prepare biocompatible RC MPs capable of prolonged release of RC. Exposure of such MPs to light radiation utilized in PDT using RC is accompanied by the generation of singlet oxygen and a cytotoxic effect on tumor cells. The RC MPs are suitable for use in local PDT of tumors. The systemic application of such RC MPs will require the inclusion of additional components in their composition, which give the RC MPs properties that promoting to their accumulation in the lesioned region of the body.

## Ethical Issues

Not applicable.

## Conflict of Interest

The authors declared no conflicts of interest.

## Acknowledgments

The work was partially supported by the grants of the Russian Foundation for Basic Research #18-29-04065, #19-515-06010, #19-33-70075, #20-04-60552 and by the Ministry of Science and Higher Education of the Russian Federation (agreement # 075-00337-20-03, project FSMG-2020-0004).
